# The Usages and Potential Uses of Alginate for Healthcare Applications

**DOI:** 10.3389/fmolb.2021.719972

**Published:** 2021-10-06

**Authors:** M. Z. I. Mollah, H. M. Zahid, Z. Mahal, Mohammad Rashed Iqbal Faruque, M. U. Khandaker

**Affiliations:** ^1^ Space Science Centre (ANGKASA), Universiti Kebangsaan Malaysia, Bangi, Malaysia; ^2^ Institute of Radiation and Polymer Technology, Bangladesh Atomic Energy Commission, Dhaka, Bangladesh; ^3^ Centre for Applied Physics and Radiation Technologies, School of Engineering and Technology, Sunway University, Selangor, Malaysia

**Keywords:** alginate, biomaterials, healthcare applications, tissue engineering, wound healing

## Abstract

Due to their unique properties, alginate-based biomaterials have been extensively used to treat different diseases, and in the regeneration of diverse organs. A lot of research has been done by the different scientific community to develop biofilms for fulfilling the need for sustainable human health. The aim of this review is to hit upon a hydrogel enhancing the scope of utilization in biomedical applications. The presence of active sites in alginate hydrogels can be manipulated for managing various non-communicable diseases by encapsulating, with the bioactive component as a potential site for chemicals in developing drugs, or for delivering macromolecule nutrients. Gels are accepted for cell implantation in tissue regeneration, as they can transfer cells to the intended site. Thus, this review will accelerate advanced research avenues in tissue engineering and the potential of alginate biofilms in the healthcare sector.

## Introduction

**FIGURE 1:**
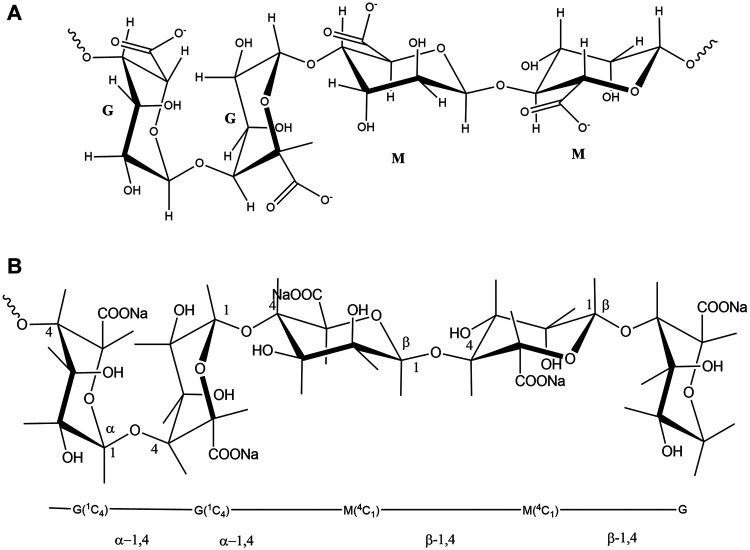
**(A)**: Chemical structures of alginate, indicating two acid groups, **(B)**: the linkage and bonding of mannuronic and guluronic groups.

**FIGURE 2:**
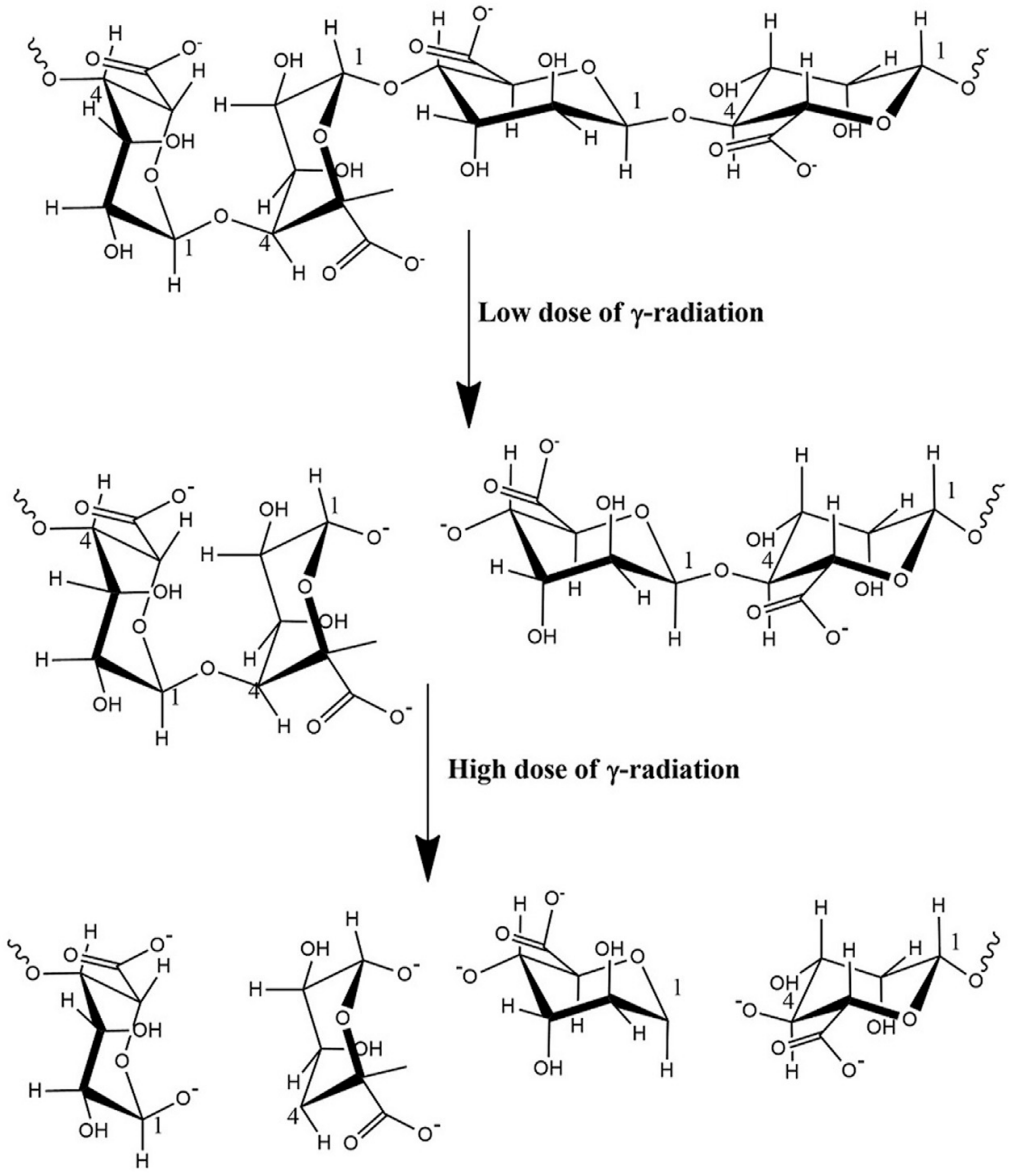
Subunits of alginate after irradiation with low and high doses of γ-radiation.

Alginate is a natural polymer, an edible hetero-polysaccharide, abundantly available in brown seaweed (*Phaeophyceae*) in nature. Natural polymers can be defined as materials that widely occur in nature, derived from a wide range of sources, extracted from plants, animals, and micro origins. Natural polymers include proteins and nucleic acid that occur in the human body, cellulose, natural rubber, silk, and wool. Modified natural polymeric materials have been used in pharmaceuticals, tissue regeneration scaffolds, drug delivery, and imaging agents (Draget and Taylor, 2011; ter Horst et al., 2019). They are essential to daily life, which is widely accepted by researchers due to their versatile applications. Alginate possesses a great potential in biomedical applications for its biodegradability and biocompatibility. Especially, in wound care, alginates are used as dressings for acute or chronic wounds and as regeneration templates. Industrially available alginate is typically extracted from the brown algae by treating with sodium hydroxide (NaOH), then filtered to accelerate the formation of alginate. The water-soluble sodium alginate is developed through conversion and purification (Peteiro, 2017). Alginates consist of linear anionic polysaccharide polymer of β-(1-4)-D-mannuronic (M-blocks) and α-l-guluronic acid (G-blocks) (Figure 1) (Lee and Mooney, 2012). In some cases, radiation can break down alginates into smaller units of molecules (M-blocks and G-blocks), as degradation produces low molecular weight substances from natural polymers (Figure 2). Alginate exhibits gelling properties due to the abundance of GM blocks and their interchain interactions.

Alginate has also some special properties: it is non-toxic, biocompatible, biodegradable, bio-stable, hydrophilic nature, which are deemed as very promising aspects for biomaterials in various state-of-the-art applications in the clinical field (Lee and Mooney, 2012). The unique criteria can also form adduct with various metal ions through electrostatic, ionic and, covalent interactions. For instance, the cationic exchanges generate an egg-box model structure through the interactions of G-blocks of alginate and electrolytic calcium (Goh et al., 2012). The binding nature to calcium has been widely used to cross-link as a bulk alginate or co-polymer in advanced applications of healthcare (Cathell and Schauer, 2007). Since alginates are promising in the field of tissue engineering due to their biocompatibility with human tissues, they are now extensively used in the control and regeneration of tissues in medical fields (Sun and Tan, 2013). Alginates can be transformed or converted into biomaterials (such as biofilms, hydrogels, foams, wafers, and fibers) to sustain a moist environment to enhance the recovery rate of wound healing. Additionally, the structure-activity relationships of alginate in biomaterials have a strong effect on the measured distribution of different drugs (antibiotic) in wound dressing materials. As a result, alginate biopolymers are exploited in various types of biomedical products, including dressing materials for skin wounds to accelerate healing properties. To establish sustainable, better quality wound dressing components, in some cases alginate-based composite materials along with wound-resistant microbes are to be used.

The focus of polymer scientists is on the progress of high-performance bio-polymeric materials. New and updated thinking has led to the demand for the scientific community to build ecological, bio-inspired, and hybrid materials that work differently. Since alginates and their derivatives possess a good water absorption capacity, they can be devoted as low viscosity emulsifiers, and shear-thinning thickeners in particular molecular weight distribution. They also hold gels forming capability due to interactive binding between G-blocks, which offer a three-dimensional “egg-box model” (Lee and Mooney, 2012; Urtuvia et al., 2017). A cross-linker to alginate, CaCl_2_ is frequently used as it has an affinity to form gel, termed as the egg-box model of cross-linking. Furthermore, CaCl_2_ salt can be applied to investigate the gelation of alginate to understand its gelation properties, morphological characteristics, molecular interaction and, structural information. Having said that, the G-enriched sample can form a hard and brittle hydrogel, while the M-enriched sample creates soft and elastic gels in the presence of the calcium ions. The protein-based Glucono-δ-lactone (GDL) is recurrently used by other agents to ionically cross-link alginate. In cutting-edge technologies, gel preparation is performed through the direct addition of the gradually hydrolyzing matrix-like as GDL to the alginate solution. The process of a sol-gel point is used to observe the power law of dynamic moduli at the critical gel. Advanced studies on gelling properties have been performed to understand the structure-function relationship of alginate blocks (Bjarnsholt, 2011; Larsen et al., 2015; Urtuvia et al., 2017). Linear polysaccharides (such as alginate) that possess a high degree of physicochemical heterogeneity influence their quality and determines potential applicability. It has a unique formulation, factors responsible for their viscosity, sol/gel transition, and water uptake ability, and dissolves in water (Szekalska et al., 2016a). The alginic acid residues of alginate can be arranged in homogenous (poly-G, poly-M) or heterogeneous (MG) block-like patterns (Venkata Prasad et al., 2012). The length of the alginic acid residues, the ratio of M and G residues, and its properties depend on the desired functions of the alginate (Vara Prasad et al., 2016). The M-enriched blocks in alginate are successfully used in the treatment of chronic wounds for their cytokine-producing capability through the human monocytes (Jung Il et al., 2020).

Based on this literature, these reviews aim to describe the phenomena of irradiated alginate-based biofilms (consisting of 5% CMC = carboxymethylcellulose, 5% PVA = polyvinyl alcohol) generated using *E. coli* and *S. typhimurium*, along with the application of biofilms in the management of different conditions like wound healing, bedsores, psoriasis, etc. In this perspective, the research is to be continued to clarify the phenomena and the general properties of biofilms. Moreover, cross-linking will be carried out at the γ-radiation plant in the Institute of Radiation and Polymer Technology (IRPT), Bangladesh Atomic Energy Commission. The preparation, characterization, modification, and degradation properties of the biofilms along with the microbiological tests were being investigated at the IRPT.

## Methods of Modification, the Alginate-Based Materials for Biomedical Application

Alginate is used as a raw material for preparing the biomaterials following multiple steps which are discussed below.

### Extracellular Film Formation

**FIGURE 3:**
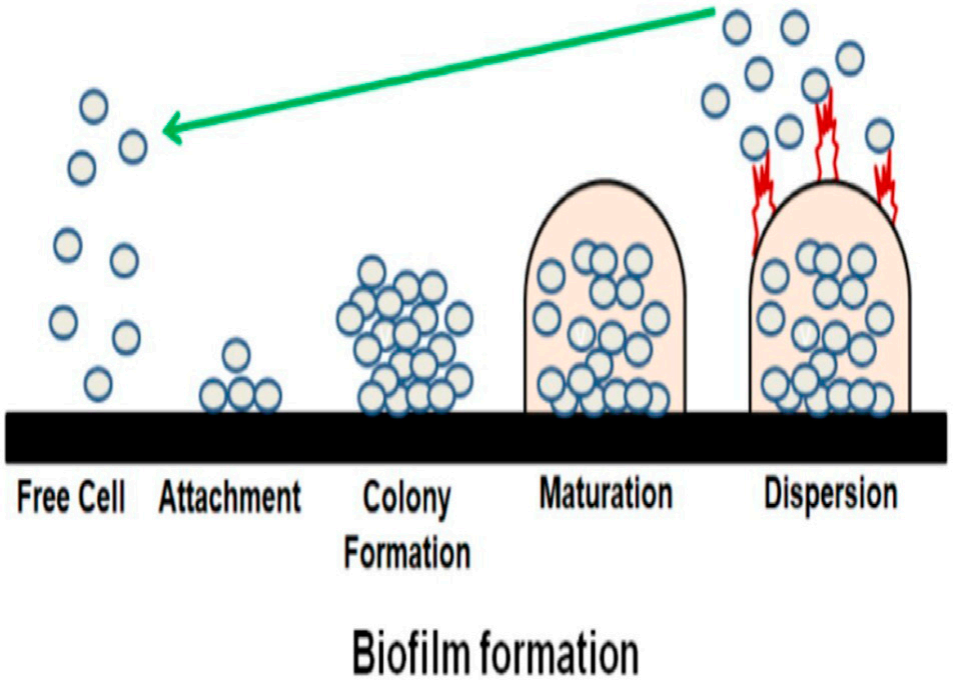
A systematic process of biofilm formation.

**FIGURE 4:**
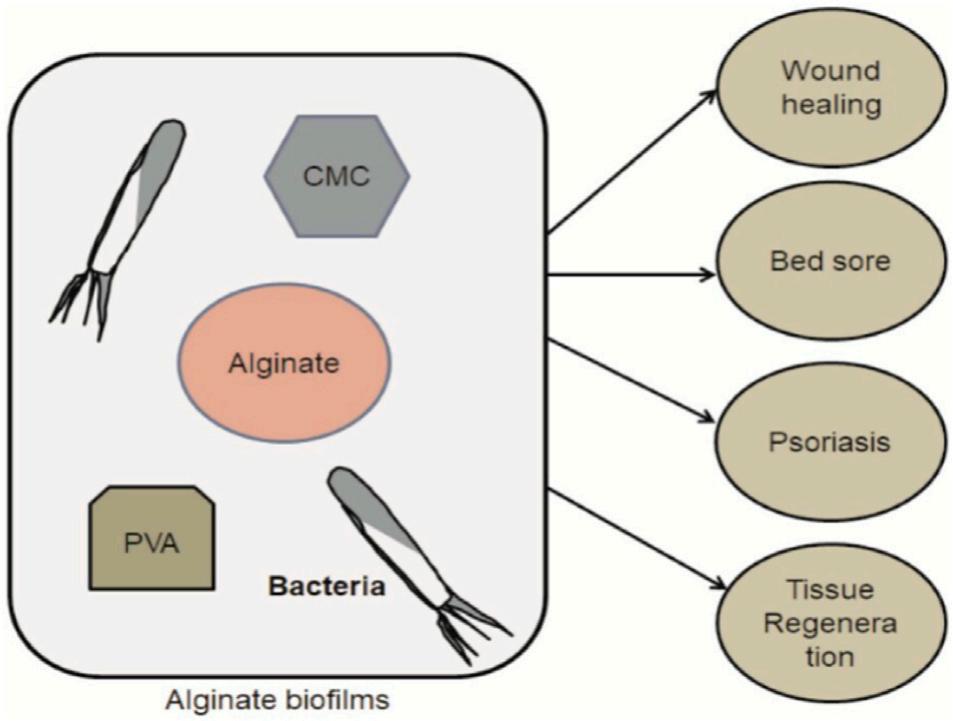
The simple steps of Alginate biofilms preparation (CMC- and PVA-based) with the target group and their proposed application in a field of healthcare.

The formation of biofilm is an intricate multistep system that secretes a combination of extracellular polymeric substance (EPS), polysaccharides, proteins and, fatty acids (Fux et al., 2003; Oppenheimer-Shaanan et al., 2013). Approximately 80% of EPS in almost all biofilms play a predominant role in maintaining and developing the biofilm. The EPS is composed of water channels to serve nutrients and oxygen to protect bacteria from the host and antibiotics (Lebeaux et al., 2013; Wei et al., 2015; Chang, 2017). Different proteins (proteases, nucleases) promote EPS production and the formation of biofilm in staphylococcal bacteria, binding proteins responsive to glucan promote EPS growth in streptococcal bacteria, while (Joo and Otto, 2012; Chao et al., 2014; Büttner et al., 2015; Paharik and Horswill, 2016) the extracellular DNA is engaged in the communication from cell to cell in *P. aeruginosa*, *staphylococcus*, and *streptococcus* at the primary stage of biofilm formation (Karatan and Watnick, 2009; Grande et al., 2014). Although the formation of biofilm is almost similar among bacteria; there could be slight differences between species in rare cases (Lebeaux et al., 2013; Veerachamy et al., 2014). The process of biofilm formation (Figures 3, 4) can be categorized into four steps; (i) primary attachment or microorganism enhancing film-formation, (ii) colony formation, (iii) maturation, and (iv) dispersion (Sauer et al., 2002; Klausen et al., 2003; Gu et al., 2013; Speziale and Geoghegan, 2015; Juhlin et al., 2017; Jamal et al., 2018).

### Microorganism Enhancing Biofilm Formation/Primary Attachment

When the planktonic cells come in contact with a film, the film adheres to the cells through physical forces or through the pili or flagella of bacteria (John, 2010; Joo and Otto, 2012; Veerachamy et al., 2014; Floyd et al., 2017). The attachment marks the initial interaction between the bacterial cells and film that can be transient or reversible (in some cases) for weak interactions (Veerachamy et al., 2014; Büttner et al., 2015; Floyd et al., 2017). The attachment of biofilms to the surfaces is directed by various interactions (such as hydrophobic, steric, protein adhesion, electrostatic), and Van der Waal forces (von Eiff et al., 2005; Stoica et al., 2017; Khatoon et al., 2018). For instance, staphylococcal biofilms contain around twenty adhesive molecules facilitating the first stage of biofilm formation (initial attachment), and their adhesion to the cell for maturation (Speziale et al., 2014). Several proteins change the adherent properties of bacteria to alter the physiochemical characteristics of bacterial surfaces, e.g., fibronectin, fibrinogen, and laminin which are responsible for accelerating the adhesion properties of bacteria to the biomaterials and tissues.

### Maturation/Development of the Biomaterials

During the maturation stage, the cells that adhered to the surfaces grow and develop by interacting with each other through auto signals production, resulting in the expression of genes responsible for the formation of biofilm (Gupta et al., 2016). At the same time, bacterial cells secrete EPS to stabilize the biofilm system and to protect themselves from antibacterial agents. Three polysaccharides; alginate, pellicle (Pel), and polysaccharide synthesis locus (Psl) are released at this stage by *P. aeruginosa* to generate the strength required for the formation of the biofilm. Meanwhile, at the accumulation and combination stages, the different layers of cell clusters are formed on the surface. The conversion of micro-colonies into macro-colonies also involved EPS where cell to cell signaling and quorum sensing (QS) are observed (Veerachamy et al., 2014; Gupta et al., 2016). In general, the maturation stage includes two steps, namely (i) intercellular communication and the production of auto-inducer signaling molecules, together with (ii) the enlargement of the microcolonies. Bacteria can observe the dimension and position of the adjacent clusters during the maturation stage to help them generate clusters and facilitate the binding with the adjacent cells (Gu et al., 2013). The whole biofilm regulates the expression of genes and proteins, apart from the individuality of each cell (Drury et al., 2004). In other words, this stage includes the production of EPS, cell accumulation, chemical interactions, and QS.

### Dispersal

During dispersion, bacteria propagate, move from one region of the body to another, and spread their infections to the surroundings. This stage is mainly responsible for chronic infection and other difficult problems (Veerachamy et al., 2014). Thus, this stage is also known as metastatic spreading (von Eiff et al., 2005; Gupta et al., 2016). Due to limited resources and the accumulation of toxic pollutants, bacteria search for nutrition to survive and the cells disperse to other regions of the host’s cells to the stressed environment (Oppenheimer-Shaanan et al., 2013; Gupta et al., 2016). Small molecules (e.g., *cis*-11-methyl-2-dodecenoic acid, DSF) can be encouraged through dispersal, to stimulate auto-phosphorylation, followed by the stimulation of c-di-GMP-phospho-diesterase (c = cyclic; di-GMP = diguanylate monophosphate) and consequently the degradation of c-di-GMP. The dissolution of EPS is another important part of the dispersion, whereby the bacteria in the biofilm produce an enzyme to break the biofilm to stabilize polysaccharides and release the surface bacteria. Next, the bacterial cells usually follow two basic options, they either (i) establish more biofilms, or (ii) do not accumulate at the cell surface (Veerachamy et al., 2014; Gupta et al., 2016).

### Quorum Sensing

Quorum sensing is a specific process of the cell to control gene expression in a density-dependent manner. In QS, the envelope of bacterial cells has an important role in intercellular signaling and communication between neighboring cells to integrate the decision-making processes (Oppenheimer-Shaanan et al., 2013; Wei et al., 2015; Gupta et al., 2016). When QS occurs in a precise volume; there is a much lower number of bacteria that must be accumulated. Through sensing, the local density can be determined by the bacterial cells while the signaling molecules reach an extreme threshold value. However, the growth of bacteria in the EPS may be largely disrupted (Puiu et al., 2017). Unlike QS, quorum quenching (QQ) is the process by which bacterial communication can be disturbed. QQ is known to disassemble and improve the susceptibility of biofilm to antibiotics (Brackman et al., 2011).

## Discussions

### Cellulose-Based Materials

**FIGURE 5:**
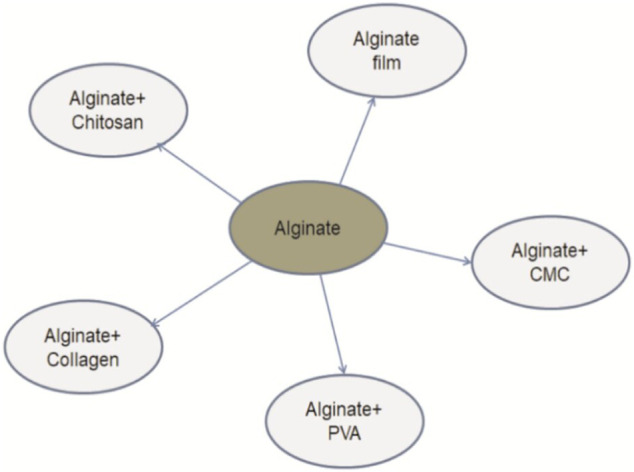
Alginate modification incorporated with different biodegradable materials.

To establish their role in healthcare applications, the preparation of biofilms using alginate with other chemicals, such as 5% CMC or 5% PVA, is performed, and subsequently encapsulated with beneficial agents. Alginate is classically utilized as a form of hydrogel for biomedical purposes for treating a wide variety of diseases together with wound healing, drug release, and biotechnology (Table 1). Cross-linked polymeric hydrogels are biocompatible for their structural similarities to certain components in the body. Moreover, hydrophilic cross-linking of polymers has been demonstrated to be obligatory to form hydrogels, whereby the cross-linking agents are vastly responsible for their physicochemical properties (Oppenheimer-Shaanan et al., 2013; Turky et al., 2021). Alginate hydrogels are prepared and modified using degradable materials at the IRPT laboratory (Figure 5). In this review, we described multiple approaches to alginate materials and their potential applications in the medical field. Table 1 describes the potential applications of hydrogels in healthcare, such as CMC, and alginate has a potential application in killing microorganisms, to be used in various burn and wound healing treatments within the healthcare sector (49, 50).

**TABLE 1:** Hydrogels derived from modifications to alginate and their applications.

Hydrogels	Applications	References
Carboxymethylcellulose (CMC), alginate, gatifloxacin	Antibacterial	(Kesavan, 2010)
Alginate-based nanocellulose	Wound-healing biotechnology	(Siqueira et al., 2019)
Bioglass/agarose, alginate	Chronic Wound-healing	(Zeng et al., 2015)
Akermanite, alginate	Wound-healing, and bio-engineering	(Yan et al., 2017)
Chitosan, alginate, alpha-tocopherol	Wound-healing	(Ehterami et al., 2019)
CMC incorporated with chitosan, alginate	Chronic wounds	(Lv et al., 2019)
Polyacrylamide, alginate, cations (Cu^2+^, Zn^2+^, Sr^2+^, Ca^2+^)	Wound-healing	(Zhou et al., 2018)
CMC hydrogel, chitosan, cellulose nanocrystal	Burn wound-healing	(Huang et al., 2018)

### Extracellular Film Formation

Free-floating and sessile, are two states of bacteria exhibiting individual features in biofilm, a wide range of genes liable for exopolysaccharide film formation and maturation, the rapid alteration in the expression resulting in the bacteria attachment on the surface of the biofilms (Bjarnsholt, 2011). Bacterial colonization subsequently initiates this transformation, where a protective blockade is consequently produced to protect the bacteria aligned with the endogenous resistance scheme of the organism or from exterior agents like antibiotics (Irie et al., 2012; Bjarnsholt, 2013; Gupta et al., 2016). Bacterial biofilms are a coherent blend of polymers with positive effects, and may be associated with a sole or numerous species of bacteria, fungi, algae, archaea, etc. (Yin et al., 2016). The microbe colony can be used to form a wide range of surfaces and living tissues, biomedical devices, drinkable water producing systems for industry, or stable aquatic systems. Water (97%) comprises a major portion of biofilms and plays a pivotal role in the flow of nutrients into a matrix of biofilm (Flemming et al., 2016). Generally, a greater portion of the whole biofilm (65–95%) consists of extracellular polysaccharides, while the remaining portion comprises microorganisms with polysaccharides and proteins being the core ingredient of biofilms (Mosharaf et al., 2018). An example of expolysaccharide generated by bacteria is alginate, where the co-deposition of bacteria along with alginate forms an experimentally compliant mold of bacterial biofilm. The development and remediation of the adverse effects of biofilms have been broadly discussed by many scholars.

### Cross-Linking

The main process in the preparation of hydrogels is the need to blend the solution which incorporates cross-linking cations (Ca^2+^) from the aqueous alginate. The Ca^2+^ can be dissociated or released from CaCO_3/_ alginate blend by adding GDL, which gradually initiates gelation and biofilm formation. These cations bind exclusively to G-blockiness as a structure of the G-blocks which concedes a significant degree of coordination of divalent ions. The G-blocks of one polymer forms junctions coordinated with another unit (G-blocks) of adjacent polymeric chains; it is fascinating in a gelling structure. The short stability in the physiological situation acts as a limitation of cross-linked alginate hydrogels. To control the gelling properties the rate of gelation is an important factor to be considered (Grant et al., 1973; Kuo and Ma, 2001; Crow and Nelson, 2006).

### Cell Cross-Linking

When cells are added to an amino-acid-based solution of arginine-glycine-aspartate (RGD)-modified alginate from a distinct dispersion, a cross-linked network structure is subsequently generated with explicit receptor-ligand interactions (Lee et al., 2003a). Non-modified alginate solution with cells aggregates forms an irregular structure. The gelation characteristic is very unstable, hence frequently manifests reversible behavior. Whenever the gel structure is broken down by a shear force, the cross-structure is regenerated immediately within very few minutes. The breakdown occurs due to the weak and reversible ligand-receptor inter-action, which is ideal for cell delivery in biotechnology especially in cell culture engineering because the gel is required to flow like a fluid in order to be injected into the body. Moreover, cell technology can offer further mechanical integrity to RGD-alginate gels by cross-linking added Ca-ions, randomly generating binding interaction between the coupling cells and adhesion ligands to the alginate interchain association (Drury et al., 2005).

### Covalent Cross-Linking Process

The physical properties of gels can be improved by comprehensively assessing their covalent cross-linking in a wide range of applications through gene engineering. The mechanical properties of alginate-based hydrogels are first scrutinized by Covalent cross-linking of alginate with polyethylene glycol (PEG) (Eiselt et al., 1999). The mechanical and swelling properties of alginate hydrogels can be firmly controlled using verities of cross-linking molecules, and by regulating the cross-linking densities. The degradation rates, along with mechanical stiffness rather than bi-functional crosslinking molecules, can be regulated through the use of a multi-functional crosslinking agent to form hydrogels. Hence, based on physico-mechanical properties, the reduction polyaldehydeguluronate (PAG) gels can be oriented with poly (acrylamide-co-hydrazide)/PAH as a multi-functional cross-linker or adipic acid dihydrazide/AAD as a bi-functional cross-linker through *in vitro* monitoring. It is observed that PAG/PAH gels possess higher mechanical stiffness before reduction and degraded more slowly than PAG/AAD gels (Lee et al., 2004). This approach is intriguing where the photo cross-linking to gelation exploits cross-linking. By using the appropriate photo (chemical) initiator, photo cross-linking may be an exciting approach to gelation that exploits covalent cross-linking. Although photo cross-linking can be carried out in gentle reaction conditions, it can still indirectly make contact with drugs and cells. Alginate forms a clear, flexible hydrogel when modification occurs with methyacrylateand cross-linked in optimum conditions, for instance, exposed to a laser for 30 s (514 nm, argon-ion laser) in the presence of eosin and tri-ethanolamine. As photo cross-linking reactions are harmful to the body, they can be partially modified using polyallylamine with α-phenoxycinnamyldieneacetyl chloride creating a new approach for photo cross-linking reactions that does not release any toxic byproducts (Sun and Tan, 1972).

### Thermal Gelation Process

**FIGURE 6:**
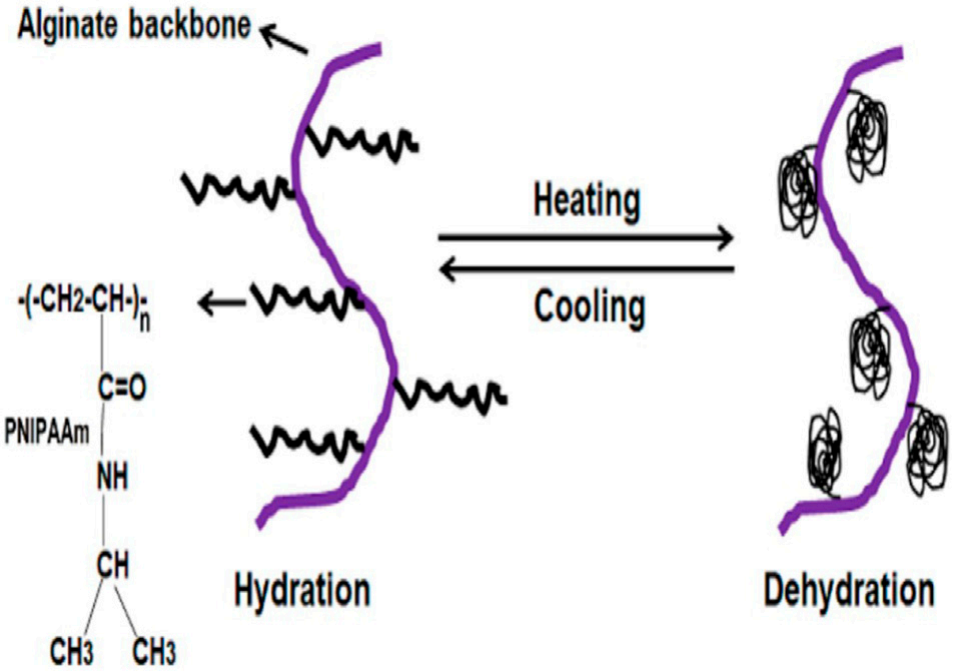
Thermal gelation process in a protein medium.

The gel formation mechanism can be described by the thermo-responsive phase transition in response to external temperature for preparing fluidic scaffold (Jinchen and Huaping, 2013). The released water molecules bind to the isopropyl group, consequently increasing inter-and intra-molecular hydrophobic interactions. As a result thermosensitive alginate hydrogel was achieved by incorporatingpoly-N-iso-propylacrylamide (PNIPAAm) and its backbone. In this synthesis, an amino-terminated NIPAAm copolymer (PNIPAAm-NH_2_) covalently coupled with carboxyl groups (-COOH) of alginate involving water-soluble carbodiimide chemistry. The temperature-dependent behavior of PNIPAAm-g-alginate hydrogels showed a remarkable decrease in the swelling ratio above 32°C (Figure 6).

A wide range of investigation in many drug delivery applications used thermosensitive hydrogels because of their swelling properties, which relies foremost on temperature changes to release the drugs to be delivered by the gels (Roy et al., 2010). The most widely exploited thermosensitive gels include the PNIPAAm hydrogels in aqueous media, where it undergoes a reversible phase transition in normal body temperature. At a constant temperature, the swelling ratio is increased, but decreases in these gels when the temperature increases. The mechanical strength and cumulative release can improve bovine serum albumin (BSA) from the gels, using sodium alginate in the semi-interpenetrating polymer network (semi-IPN) structure indicate diversity and applicability of hydrogel in the drug delivery system (Zhao et al., 2010).

### Microorganism Enhancing

Planktonic cells and, biomaterial microbes play an advantageous role in biomedical applications due to their forbearance to inert materials and ecological anxiety. In unicellular microorganisms, the symbiotic correlation with co-existing microorganisms confides in essential elements from a poly-cellular constituent. A typical exemplification of this symbiosis is the *Escherichia* sp. located in the lower intestine of humans and other warm-blooded animals. *E. coli* sp. can be commensal, existing in a symbiotic state and providing resistance against pathogenic organisms (Mohammad, 2010; Bjarnsholt, 2013; Rimondini et al., 2016). A biofilm is a well-organized formulation of polymers produced by microorganisms to control minerals and other essential nutrients. It can also be associated with unique or related species of bacteria, algae, fungi, and archaea. Based on the environmental conditions, the formation of biofilm can diversify its structural and elemental modifications (Yin et al., 2016). Based on previous studies, a colony of bacteria, fungi, and algae have been employed for neutralization, degradation, mineralization, and to cut out adequate inorganic/organic pollutants from an adulterated or contaminated atmosphere. Among the different processes, it has been shown that a biofilm-mediated healthcare facility has been contemplated as an eco-friendly and low-cost choice. Bioremediation is a technology that utilizes microbes to decontaminate and degrade different pollutants into less toxic products. Bioremediation was favored as an eco-friendly technique compared to the conventional facilities for the detoxification of toxic elements (Balaji et al., 2014; Mitra and Suman, 2016; Sfaelou et al., 2016; Miranda et al., 2017).

### Biodegradation

Ionically cross-linked alginate gels can be dissolved by releasing divalent ions into the surrounding media due to the exchange reactions with monovalent cations such as sodium ions. Sodium ions are monovalent cations and can be ionically cross-linked in alginate gels to release divalent ions which can be dissolved into the surrounding media due to the exchange reaction has occurred among the ions and cations (Al-Shamkhani and Duncan, 1995). Degradation of alginate in physiological conditions, which is a lucrative approach, can be obtained by partial oxidation, which does not interrupt the properties of the gel. Alginate can be degraded and indicated by slight oxidation, the latent drug delivery avenue in cells and drugs for diversified applications in healthcare. Alginate is usually oxidized using sodium periodate as it undergoes periodate oxidation that cleaves the *cis*-diol group into the uronate residues present in alginate and degrades the backbone of alginate. The blockiness of G-blocks and the partial oxidation of the G-block allow the formation of a degradable gel. Only polyguluronate (PG) oxidized as sodium periodate to prepare PAG can covalently be cross-linked with AAD during ionic cross-linking polymerization to form a gel. The reaction occurs quickly between the aldehydes and hydrazides group, as hydrazone bonds are quite vulnerable to hydrolysis which promotes the degradation of the gels in an aqueous solution (Lee et al., 2000).

## Applications of Alginates

Alginates are used to coat fruits and vegetables; as a microbial and viral protection by-product; and as a gelling, thickening, stabilizing, or emulsifying agent in food factories (Gheorghita Puscaselu et al., 2020). Whereby, alginate substantially plays a vital role in the sustainable release of drug delivery products in the pharmaceuticals industry. In this review, the controlled drug delivery system of alginate or its derivatives are depicted in various clinical aspects (Figure 4).

### Pharmaceuticals and Therapeutic Clinical Observation

Alginate also possesses raft‐forming properties like Gaviscon Liquid (GL), containing sodium alginate, CaCO_3_, NaHCO_3_, and flourishes a robust floating raft in the bitter domain of the abdomen (Yousaf et al., 2019). The alginate-based hydrogels are frequently associated with therapeutics of other origins such as antacids to amplify the competence of raft formulations (Mandel et al., 2000). Alginate protects the bacteria and enhances adhesion to solid surfaces, whereby, the adhesion induces the migration of alginate-based bio-synthetic genes and increases the production of alginate. Thus, alginate biofilms are developed to facilitate the survival and growth of bacteria. These biofilms manifest high water absorption and low viscosity emulsification at a low molecular weight (50–2000 kDa) (Mangwani et al., 2016).

Clinical investigations have been conducted as an anti-AIDS drug in China, where heparinoid alginate derivatives were explored for the treatment of HIV. They used sulfated enriched mannuronic and guluronic heterogeneous alginate fragments incorporated with heparinoid polysaccharides, drug 911 which consists of 10 kDa to 1.5 sulfates and 1.0 carboxyl groups per sugar residue. It was diagnosed that heparinoid 911 interacted with the positively charged regions of glycoproteins present on the cell surface, leading to shielding effect on these regions, thus counteracting HIV-virus binding to the cell surface. The mode action of 911 was found to be related to the inhibition of viral reverse transcriptase and prevention of viral adsorption. A significant inhibitory effect on DNA polymerase of the hepatitis B virus was reported, meaning it can be applied in hepatitis B treatment (Xin et al., 2000; Wu et al., 2011; Szekalska et al., 2016b).

Alginate consisting oligosaccharides caused lower blood pressure, it appears that the hypertensive mechanism is associated with calcium antagonist activity toward voltage-operated calcium channels. It was clinically reported that sodium alginate in a dose of 60 mg/day decreased blood pressure eliminated hypertension after 2 weeks of treatment. Another trial revealed that polymers’ facility prevented early stage kidney injury by decreasing the rate of glomerular filtration. Apart from this, alginate-based potassium tested as a promising agent for mitigating cardiovascular complications associated with hypertension, including cardiac and renal hypertrophy in the risk of stroke occurrence (Chen et al., 2010; Morria et al., 2013; Szekalska et al., 2016b). Alginate biopolymers are extensively used to develop various dressing materials for the treatment of wound healing (Varaprasad et al., 2020). Different cardiovascular diseases, such as atherosclerosis, myocardial infarction, etc. are the leading cause of morbidity and mortality. The alginate and hydrogels are mostly applicable in cardiac regeneration (Cattelan et al., 2020).

### Delivery of Drug Molecules

A nanoporous (pore size ∼5 nm) alginate gel is utilized in the release of various small molecular weight drugs, whereby the tiny molecules can pass rapidly through the gel (Boontheekul et al., 2005). A combination of different drugs associated with alginate-based gels are used for instantaneous and continuous release. For instance, alginate grafted with polycaprolactone is cross-linked with Ca^2+^ for sustained delivery of theophylline. The sustained release of theophylline was also obtained from alginate microspheres with carbon nanotubes (Zhang et al., 2010). Encapsulated chitosan-alginate may also be used in drug application. It is formulated as magnetic products of alginate-chitosan beads for passive targeting in the gastrointestinal tract tagged with albendazole (ABZ) (Wang et al., 2010).

### Protein Delivery

At present alginate is also widely used in protein drugs delivery systems, which can easily be integrated with alginate-based formulations through a comparatively simple process. The rate of protein delivery can be maintained in different ways from alginate globules. The flow rate of protein release from alginate gels is usually rapid. Hence, if the degradation rate is changed, the pace of protein release from the gel has to be harmonized (Silva and Mooney, 2010). The released angiogenic molecules can be controlled by the alginates gel. As a building block molecule, alginate is also used in the polymeric structure of a tetra-functional acetal-linked network by using polymer synthesis. The rapid release of gels can enhance protein-hydrogel interactions in the interchain association of alginate, whereby it is widely applied in tissue engineering and regeneration (George and Abraham, 2006).

### Wound Dressings

Wound healing treatment demands many desirable functions fulfilled by wound dressing materials prepared from alginate. Traditional dressing materials like gauze only have a barrier function, however, modern alginate dressings present a moist wound environment and ease healing (Queen et al., 2004) as it is prepared using polymerization and cross-linked in an aqueous solution with divalent cations. In ry conditions alginate dressing materials immersed in fluidic concentration re-gel transport water to the wound to maintain a damp situation and protect the wound location from bacterial infection. Available dressings on the market, include Algi-cell (Derma Sciences), Algi-Site M (Smith and Nephew), Comfeel Plus (Coloplast), Kaltostat (ConvaTec), Sorbsan (UDL Laboratories), and Tegagen (3 M Healthcare) (Balakrishnan et al., 2006).

### Cell Culture

The alginate-based gels are used in healthcare applications especially in mammalian cell technology as a multidimensional (2-D/3-D) culture system. The RGD-adopted alginate gels are most frequently employed in vitro tissue culture engineering; whereas RGD peptides in alginate are usually exploited to regulate the interacting characteristics of myoblasts (Rowley et al., 2002). The combination of alginate backbone and RGD peptides bond enhance the propagation and adhesion of myoblast culture onto alginate gels (Rowley et al., 1999).

Immobilization of living cells persuading factors in the alginate matrix is frequently used in cell engineering. A lot of advanced research has been conducted in Australia for the development of cell transplantation therapy in long-term diabetes and neurodegenerative Immupel™. It has a selectively permeable ability to protecting via encapsulation living cells from the host immune system, managing their function and able to differentiate between them accurately. Apparently, ALG-based foodstuffs NTCELL^(R)^ and DIABECELL^(R)^ are in an advanced stage of clinical investigation (yang and Yoon, 2015; Szekalska et al., 2016b) since the last decade.

### Antibiotics

Alginate-based gel is usually used in antibiotics, while a number of drawbacks have been observed in the following processes of biofilm resistance to antibiotics:

i) In a biofilm, the change in bacterial growth causes antibiotic diffusion to differ (Fux et al., 2003; Joo and Otto, 2012).
ii) Horizontal transformation of gene resistance is acquired by *P. aeruginosa* (Gupta et al., 2016).
iii) It is responsible for resistance as multidrug efflux pumps antibiotic agents present in the system (Zhang and Mah, 2008; Hall and Mah, 2017).
iv) Recurrently, the dual-species biofilm manifests increased resistance, which is already revealed as a barrier to the antibiotic (Adam et al., 2002).

Thus, due to these limitations, biofilms are problematic to apply in current antibiotic therapies, for treatment with biofilm can cause infections, and it is recommended that state-of-the-art advancement of implantable biomedical devices be immediately explored (Singhai et al., 2012; Shah et al., 2013; Stoodley et al., 2013). Despite the shortcomings, alginate-biofilms have been exploited in the regeneration of different types of tissues (Table 2) including versatile applications in healthcare. Table 2 describes the cutting-edge applications of the alginate-based biomaterials in tissue regeneration using molecular biology related techniques. Using modern technologies, from stem cells different types of tissues including blood vessels, bone, muscles, cartilage, and nerve cells can be generated using alginate and alginate-based biomaterials (Tuan et al., 2003; Levenberg et al., 2005; Dashtdar et al., 2011; Kolambkar et al., 2011).

**TABLE 2:** Application of alginate gels in the tissue regenerations.

Gel	Activities	Tissues	Descriptions
Alginate	Tissue regeneration with protein and cell delivery	Blood vessels	The injected alginate gels into ischemic muscle tissues are beneficial for enduring release of VEGF, and configuration of VEGF gradients in close to tissues (Lee et al., 2003b)
		Bone	Modified RGD-alginate gels employed for regeneration of femoral rift in rodents with a mum dose of BM (Kolambkar et al., 2011). The controlled delivery of BMP-2 and BMP-7 via alginate gels enhanced osteogenic segregation of bone marrow resulting technology of stem cells (Buket Basmanav et al., 2008)
		Cartilage	Alginate may enhance hydrogenesis, stem cell technology (chondrogenesis) regulates the morphology of cell encapsulation (Dashtdar et al., 2011) and alginate gels promote a rounded morphology to accelerate the differentiation process of cells (Tuan et al., 2003)
		Muscle	Alginate gels facilitated the process of growth factor release, skeletal muscle reformation, stem cell transplantation etc. (Saxena et al., 1999; Levenberg et al., 2005). Alginate gels also used as stimuli-responsive for stimulating myogenesis, combined delivery of VEGF, growth factor-1 like insulin (IGF-1). The growth factor works as a significant muscle formation and regeneration when localized and sustained delivery occurred in the system (Borselli et al., 2010)
		Nerve	Investigation has been done for the remapping of the peripheral and central nervous system by the significant use of alginate gels. Extremely isotropic capillary gels, imported into acute cervical spinal cord lesions in adult rats, incorporated into the spinal cord parenchyma, and assisted axonal regrowth (Prang et al., 2006), as well as the gels of alginate, may help for cell-based neural therapies (Li et al., 2006)
		Pancreas	It is revealed that type I diabetes is curable, placing grafts of alginate gel as the transplantation of encapsulated pancreatic islet. This type of approach has succeeded in Type I diabetes in an animal model experiment without prior immunosuppressive drugs (Lim and Sun, 1980; Lacy et al., 1991; Calafiore, 2003)
		Liver	In the field of tissue engineering, alginate gels wrapping hepatocytes may bid a suitable platform for increasing a bio-artificial liver as they are easily accomplished and stored (Selden and Hodgson, 2004; Koizumi et al., 2007)

## Conclusions and Future Perspectives

In the production of natural and biosynthetic materials for medical devices, our interest has been greatly increased. Another, more in-depth, experiment should be promptly commenced to find potential applications for medical purposes. Alginates holds great potential as biomaterials for biomedical applications in human healthcare, and mainstream biotechnology like tissue plantation, drug delivery, wound healing, stem cell culture, and gene engineering. Alginate-based biomaterials can be detached with tolerable discomfort compared to other commercially available conventional wound dressing materials. Furthermore, since sustained released antibiotics prepared using alginate exhibit high absorbing capacity, they are most useful to treat deep burns. To prepare alginate derivatives with ultramodern characteristics, the most effective physicochemical, mechanical, and thermal properties that need to be improved for these applications include biocompatibility, mild gelation, and moderate modification of alginate gels. Finally, the world is developing; progressing through newly invented technologies and high-tech facilities, but accomodation in these sectors for the environment and many other aspects of sustainability are still limited. Hence, modification and characterization of alginate polymers and their biofilms in biomedical application for various purposes could play a pivotal factor in its development for the medical sector, along with minimizing environmental effects to allow for sustainable societies. The knowledge acquired from the study can evolve the potentiality in healthcare application in the medical sector.
