# Supplementary material for: The Usages and Potential Uses of Alginate for Healthcare Applications
Source: Front Mol Biosci. 2021 Oct 6;8:719972. doi: 10.3389/fmolb.2021.719972 (PMC8530156; doi:10.3389/fmolb.2021.719972)
Supplement: Supplementary file 1 [file Table1.docx]

**Table 1. Hydrogels derived from modifications to alginate** and their application

| **Hydrogels** | **Applications** | **References** |
| --- | --- | --- |
| Carboxymethylcellulose (CMC), alginate, gatifloxacin | Antibacterial | Kesavan*et al*., 2010 [46] |
| Alginate- based nanocellulose | Wound-healing biotechnology | Siqueira*et al*., 2019 [47] |
| Bioglass/agarose, alginate | Chronic Wound- healing | Zeng, Han, Li, & Chang, 2015 [48] |
| Akermanite, alginate | Wound- healing and bio-engineering | Yan et al., 2017 [49] |
| Chitosan, alginate, alpha-tocopherol | Wound-healing | Ehterami*et al.,* 2019 [50] |
| CMC incorporated with chitosan, alginate | Chronic wounds | Lv*et al*., 2019 [51] |
| Polyacrylamide, alginate, cations (Cu^2+^, Zn^2+^, Sr^2+^, Ca^2+^) | Wound-healing | Zhou *et al*., 2018 [52] |
| CMC hydrogel, chitosan, cellulose nanocrystal | Burn wound-healing | Huang *et al*., 2018 [53] |
