# Supplementary material for: The Usages and Potential Uses of Alginate for Healthcare Applications
Source: Front Mol Biosci. 2021 Oct 6;8:719972. doi: 10.3389/fmolb.2021.719972 (PMC8530156; doi:10.3389/fmolb.2021.719972)
Supplement: Supplementary file 2 [file Table2.docx]

Table 2. Application of alginate gels in the tissue regenerations.

| **Gel** | **Activities** | **Tissues** | **Descriptions** |
| --- | --- | --- | --- |
| **Alginate** | Tissue regeneration with protein and cell delivery | Blood vessels | The injected alginate gels into ischemic muscle tissues are beneficial for enduring release of VEGF, and configuration of VEGF gradients in close to tissues [106]. |
|  |  | Bone | Modified RGD-alginate gels employed for regeneration of femoral rift in rodents with a mum dose of BM [103]. The controlled delivery of BMP-2 and BMP-7 via alginate gels enhanced osteogenic segregation of bone marrow resulting technology of stem cells [107]. |
|  |  | Cartilage | Alginate may enhance hydrogenesis, stem cell technology (chondrogenesis) regulates the morphology of cell encapsulation [104] and alginate gels promote a rounded morphology to accelerate the differentiation process of cells [108]. |
|  |  | Muscle | Alginate gels facilitated the process of growth factor release, skeletal muscle reformation, stem cell transplantation etc. [109, 105]. Alginate gels also use as stimuli responsive for stimulating myogenesis, combined delivery of VEGF, growth factor-1 like insulin (IGF-1). The growth factor works as a significant muscle formation and regeneration when localized and sustained delivery occurred in the system [110]. |
|  |  | Nerve | Investigation has been done for the remapping of peripheral and central nervous system by the significantly use of alginate gels, it is extremely isotropic capillary gels, imported into acute cervical spinal cord lesions in adult rats, incorporated into the spinal cord parenchyma and assisted axonal regrowth [111] as well as the gels of alginate may help for cell-based neural therapies [112]. |
|  |  | Pancreas | It is revealed that type I diabetes is curable, placing grafts of alginate gel as the transplantation of encapsulated pancreatic islet. This type of approaches has succeeded in Type I diabetes in a animal model experiment without prior any immunosuppressive drugs [113, 114, 115]. |
|  |  | Liver | In the field of tissue engineering, alginate gels wrapping hepatocytes may bid a suitable platform for increasing a bio-artificial liver as they are easily accomplished and stored [116, 117]. |
